# Therapeutic potentials of resveratrol in combination with radiotherapy and chemotherapy during glioblastoma treatment: a mechanistic review

**DOI:** 10.1186/s12935-021-02099-0

**Published:** 2021-07-21

**Authors:** AmirAhmad Arabzadeh, Tohid Mortezazadeh, Tayebeh Aryafar, Esmaeil Gharepapagh, Mehrsa Majdaeen, Bagher Farhood

**Affiliations:** 1 Department of Surgery, School of Medicine, Ardabil University of Medical Sciences, Ardabil, Iran; 2 Department of Medical Physics, School of Medicine, Tabriz University of Medical Science, Tabriz, Iran; 3 Department of Radiation Sciences, Yasuj University of Medical Sciences, Yasuj, Iran; 4 Medical Radiation Sciences Research Team , Tabriz University of Medical Science, Tabriz, Iran; 5 Department of Radiotherapy and Oncology, Razi Hospital, Guilan University of Medical Sciences, Rasht, Iran; 6 Department of Medical Physics and Radiology, Faculty of Paramedical Sciences, Kashan University of Medical Sciences, Kashan, Iran

**Keywords:** Resveratrol, Glioblastoma, Radiation therapy, Chemotherapy

## Abstract

Glioblastoma, WHO grade IV astrocytoma, is the most aggressive type of brain tumors. These cancerous cells have a rapid growth rate, tendency to penetrate vital brain structures, molecular heterogeneity, etc. and this cancer is associated with a poor prognosis and low survival rate. Due to the resistance of glioblastoma cells to conventional therapeutic modalities (such as radiation therapy and chemotherapy) as well as the adverse effects of these modalities, the researchers have attempted to discover an appropriate alternative or adjuvant treatment for glioblastoma. Resveratrol, as an herbal and natural polyphenolic compound, has anti-tumoral property and has shown to be effective in GBM treatment. Resveratrol exerts its anti-tumoral effect through various mechanisms such as regulation of cell cycle progression and cell proliferation, autophagy, oxidant system, apoptosis pathways, and so on. Resveratrol in combination with radiation therapy and chemotherapy has also been used. In the present study, we summarized the current findings on therapeutic potentials of resveratrol in glioblastoma radiotherapy and chemotherapy.

## Introduction

Gliomas arise from glia cells (including oligodendrocytes, astrocytes, ependymal cells) or cancer stem cells and are categorized histologically in accordance with the similarity to their putative cell of origin [1]. They are different in terms of aggressiveness from benign to highly malignant which are graded from I to IV by the World Health Organization (WHO) [2, 3]. The most frequent of these fatal tumors in adults is WHO grade IV astrocytoma or glioblastoma (or glioblastoma multiform (GBM)) which occurs in brain or spinal cord and accounts for 50% of diffuse gliomas [4, 5]. The standard treatment modality for GBM is complete surgical resection followed by radiation therapy and chemotherapy. Moreover, a number of efforts have been made to develop new therapeutic modalities. Despite such efforts, these treatment modalities do not dramatically improve clinical outcome of GBM patients [3, 5, 6]. According to a report by Jackson et al. [2], rapid progression, resistance to treatment, and inexorable recurrence of GBM can be attributed to some factors such as its rapid growth rate, its tendency to penetrate vital brain structures, its molecular heterogeneity, problems in obtaining high concentrations of chemotherapeutic drugs in the central nervous system, etc. It has also been reported that dysregulation of cellular signaling pathways (such as hyperactivation of PI3 kinase pathway) and genetic mutations (such as mutation in retinoblastoma and p53 genes) can play critical roles in GBM ignition, invasion and progression [7–9].

The use of radiation therapy and chemotherapy can also damage the normal tissues severely; as it may stop the course of cancer treatment due to acute reactions [10]. In other words, these two treatment modalities can be considered as double-edged swords; as the use of these treatments may induce second cancers and adverse effects, negatively affecting patients’ quality of life. In this regard, management of early and late complications arisen from cancer treatment without a negative effect on cancer response is one of the most important goals in radiation therapy and chemotherapy [11, 12]. Therefore, the introduction and development of radio/chemotherapeutic modifiers (as radio/chemoprotectors and radio/chemosensitizers (can improve therapeutic efficiencies of chemotherapy and radiotherapy [13].

**Fig. 1:**
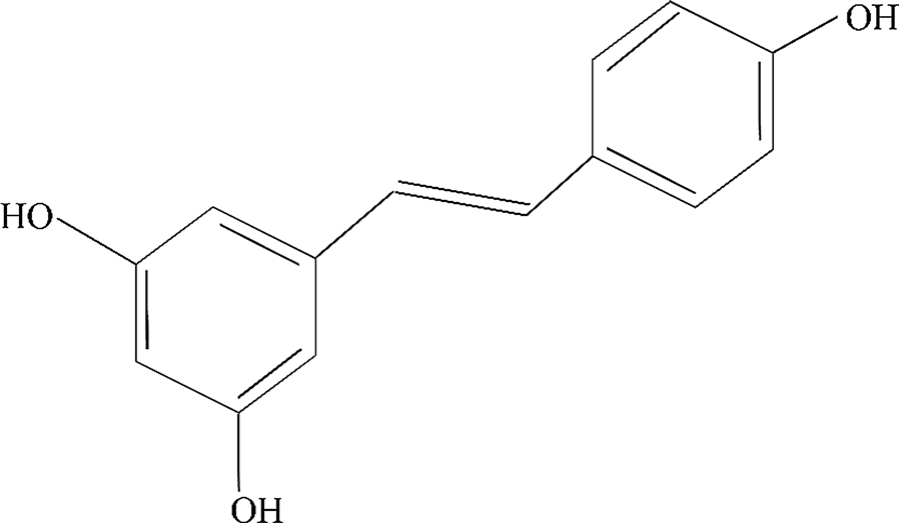
Chemical structure of resveratrol

During past decades, tendency to use herbal and natural compounds or their derivatives (with less toxicities) have attracted much attention for various therapeutic purposes, especially cancer treatment. Resveratrol (3,5,4′-trihydroxy-*trans*-stilbene) is a herbal and natural polyphenolic compound that can be found in grapes, red wine, peanuts, soy, etc. [14–16]. The molecular structure of resveratrol is shown in Fig. 1. This herbal agent has some abilities to kill cancerous cells and amplify tumor response to therapeutic modalities such as radiotherapy and chemotherapy [17]. Interesting properties of resveratrol as a potential anti-oxidant agent have been resulted to its use in other different health benefits, such as neuroprotective and radioprotective effects [18]. Other biological activities of resveratrol such as cardioprotective, chemopreventive, anti-inflammatory, proapoptotic, and anti-proliferative properties have also been reported [15, 16].

Despite the remarkable beneficial effects of resveratrol, some studies have failed to reflect these properties which it may be due to its high absorption but low bioavailability [19, 20]. Moreover, the use of resveratrol can be compromised because of its hydrophilicity when apply in lipophilic systems [21]; nevertheless, this drawback could be overcome by structural modification [22]. In this regard, some researchers have developed novel resveratrol derivatives, such as pterostilbene [23, 24], trimethoxystilbene [25, 26], hydroxystilbene [27, 28], dihydroxystilbene [29, 30], bridged stilbenes [31], etc. Compared to resveratrol, modifying some substitutions can improve their bioavailability and biological activities.

The use of nanotechnological strategies can improve the bioavailability and efficacy of resveratrol and its analogues. In this regard, some studies have been investigated the efficacy of resveratrol delivery systems on treatment of many tumors [32–35].

The present review aims to summarize current studies on therapeutic potentials of resveratrol in GBM radiotherapy and chemotherapy. It is also tried to present the resveratrol roles and molecular mechanisms involved in GBM radiotherapy and chemotherapy. Furthermore, the findings obtained from derivatives/analogues and delivery systems of resveratrol in GBM treatment have been addressed.

## Role of resveratrol in glioblastoma treatment

Products derived from nature have been one of the most main and considerable sources in drug discovery and development [36–38]. For many years, the use of natural products and/or natural herbal formulations has been of interest to humans for the preservation of health, improvement of physical and mental health, and prevention of diseases. Many studies have also reported that some natural products in combination with radiotherapy and chemotherapy can have radio/chemoprotective and/or synergistic effects in terms of alleviating cancer radiotherapy/chemotherapy associated complications and increasing the therapeutic efficacy [14, 39]. Moreover, some of them can penetrate blood brain barrier (BBB) which this property is one of the principal consideration for development of drugs for central nervous system (CNS) [40, 41].

Resveratrol is a natural pharmaceutical compound which has a broad range of biological activities such as anti-fungal, anti-viral, anti-inflammatory, anti-oxidant, and anti-aging effects [42–46]. The biological activities of this natural polyphenol are mainly attributed to its unique structure feature with multiple phenolic hydroxyl groups; as polyphenol components are able to scavenge free radicals to produce more stable molecules with low toxicity than the original radicals [47]. It has also been reported that resveratrol can prevent the tumor initiation, promotion and progression [48]; for example, its anti-tumoral activity has been assessed in many tumor types, such as colorectal, prostate, lung, liver, breast cancers, etc. [49–52]. Furthermore, resveratrol can cross the BBB successfully and hence, it may be used as an efficient therapeutic or protective agent against CNS-related injuries/disorders and tumors, including global cerebral ischemic injury [53], Alzheimer’s [54–56], Parkinson [57, 58] and GBM [59, 60].

Some studies have also showed that resveratrol, as a radio/chemosensitizer agent, can enhance the therapeutic efficacy of radiotherapy/chemotherapeutic drugs against glioblastoma cells which are discussed the following (Table 1).

**Table 1:** Anti-tumoral effects of resveratrol in glioblastoma radiotherapy/chemotherapy

Model	Cell line(s)	Resveratrol dosage; route of administration	Exposure conditions of RT	Chemotherapeutic drug; dosage; route of administration	Co-treatment outcomes	Refs.
In vitro	U-87MG	20 µM	5 Gy; 180 KV X-rays	–	Induction of a delay in cell cycle progression, enhancement of GJIC	[82]
In vitro and in vivo	CD133	100 µM	2, 4, 6, 8, and 10 Gy; 1.25 MeV (cobalt-60 γ-rays)	–	Induction of apoptosis, suppression of STAT3 signaling, ↑survival rate	[70]
In vitro and in vivo	SU-2	75 µmol/L (for in vitro) and 150 mg/kg/day (for in vivo); *ip*	2, 4 and 6 Gy; 6 MV X-rays	–	↑radiosensitivity, prevention of self-renewal and stemness, ↑apoptosis, induction of autophagy, inhibition of DNA repair	[101]
In vitro	U87MG	20 µM	2 Gy; 1.25 MeV (cobalt-60 γ-rays)	–	↓colony number, ↑DNA damage, ↑radiosensitivity	[100]
In vitro	DBTRG	50 µM	–	Paclitaxel; 50 µM	↑mitochondrial ROS levels, ↑activation of TRPM2 channel, ↑caspase 3 activity, ↑influx of Ca^2+^ into the cell through TRPM2 channel	[108]
In vitro	T98G	100 µM	–	Temozolomide; 100 µM	↑chemosensitivity, ↑apoptotic morphology (such as nuclear and cytoplasmic condensation and chromatin aggregation), ↑cleavage of caspase-3, ↓intracellular level and nuclear translocation of NF-κB, repression of MGMT expression	[120]
In vitro and in vivo	GIC400 andGIC411	20 and 40 µM (for in vitro) and 12.5 mg/kg/day (for in vivo); *ip*	–	Temozolomide; 200 and 400 µM (for in vitro) and 68 mg/kg/day (for in vivo); oral	↓cell viability, induction of apoptosis, activation of DSBs/pATM/pATR/p53 pathway, inhibition of self-renewal capacity and promotion of cell differentiation, inactivation of STAT3, inhibition of tumor growth	[119]
In vitro and in vivo	T98G and U138	2, 4, 8, 10, 16 and 32 µM (for in vitro) and 10 mg/kg/day; *ip*	–	Temozolomide; 400 µM (for in vitro) and 25 mg/kg//day (for in vivo); *ip*	↓cell viability and proliferation, ↑apoptosis (↑Cleaved caspase-3 and Bax, ↓XIAP and Bcl‐2), suppression of Wnt signaling pathway, downregulation of MGMT expression	[121]
In vitro	RG-2, LN-18 and LN-428	25, 50, 75 and 100 µM	–	Temozolomide; 250, 500, 750 and 1000 µM	Inhibition of growth cell, down-regulation of MGMT overexpression, ↓expression of STAT3, ↓survivin and Bcl-2 levels, Inhibition of STAT3/Bcl-2/survivin signaling pathway	[122]
In vitro and in vivo	SHG44	10 µM (for in vitro) and 40 mg/kg/day (for in vivo); oral	–	Temozolomide; 100 µM (for in vitro) and 68 mg/kg/day (for in vivo); oral	Induction of cell cycle arrest in the G2/M phase, ↑expression of GFAP, down-regulation of MMP-9 expression, inhibition of cell migration, ↑ROS production, activation of AMPK, inhibition of mTOR signaling, down-regulation of Bcl-2, ↓tumor volume, ↓Ki-67 expression	[133]
In vitro and in vivo	U87 MG	10 µM (for in vitro) and 12.5 mg/kg/day (for in vivo); *ip*	–	Temozolomide; 100–400 µM (for in vitro) and 10 mg/kg/day (for in vivo); *ip*	↓autophagy, ↑apoptosis, ↓cell viability, ↑chemosensitivity,↑cell death, ↓tumor volume, ↓ERK activity and LC3-II protein levels, ↑cleavage of PARP	[172]
In vitro	U87, U138 and U251	30 µM	–	Temozolomide; 100 µM	↑autophagy, abrogation of temozolomide-induced G2 arrest, ↑gammaH2AX, pATM and pChk2, ↑cyclin B and pRb levels, ↓pWee1 and pCdk1 levels, induction of mitotic catastrophe (aberrant chromosome condensation and mitotic phenotype, micronuclei and nuclearfragmentation, abnormal/triple mitosis, ↑percentages of irregular nuclei and large nuclei), ↓clonogenic growth, ↑senescence	[163]
In vitro and in vivo	U251MG and C6	7.5, 15 and 30 µM and 10 mg/kg/day; *ip*	–	Temozolomide; 10 mg/kg/day and thrice a week; *ip*	Inhibition of temozolomide-induced autophagy and promotion of apoptosis (up to 15 µM resveratrol), inhibition of ERK1/2-dependent autophagy	[164]

↑, Increase; ↓, Decease; GJIC, Gap junction intercellular communication; MGMT, *O*^6^-methylguanine-DNA methyltransferase; STAT3, signal transducer and activator of transcription 3; GFAP, Glial fibrillary acid protein; MMP-9, matrix metalloproteinase-9; ERK, Extracellular signal-regulated kinase; PARP, poly(ADP-ribose) polymerase; ROS, reactive oxygen species

### Therapeutic potentials of resveratrol in glioblastoma radiotherapy

Standard treatment for newly diagnosed GBM patients is complete surgical resection followed by adjuvant chemoradiation therapy (CRT) [6, 61]. Conventional radiotherapy protocol used in these patients is as follows: total dose of 59.4 to 60 Gy, dose per fraction of 1.8 or 2.0 Gy, five fractions per week and treatment period of 6 to 7 weeks [62]. In this regard, the use of resveratrol in combination with radiotherapy can increase the therapeutic efficacy (synergistic effects) [63, 64].

One of the reasons for radioresistance of tumoral cells is the upregulation of transcription factors such as signal-transducer-and-activator-of-transcriptions (STATs) after exposure to radiation [12]. These enzymes are transcription factors for cytokine signaling which are constitutively activated in some tumor types such as prostate, breast, brain cancers, nasopharyngeal carcinoma, leukemia, etc. [65–68]. It has been proposed that the targeting of STATs in combination with radiation therapy can be considered as a strategy for overcoming tumor resistance. STAT3, as one of the subfamilies, contributes in modulation of angiogenesis, suppression of apoptosis, regulation of cell cycle progression and metastasis through stimulation of VEGF, MMP-2, MMP-9 and IAP-1 [12, 69]. In a study by Yang et al. [70], the therapeutic effect of resveratrol on GBM-derived radioresistant tumor initiating cells was investigated. In their study, the cells in control and irradiated groups were categorized; as the cells of irradiated groups were exposed to various radiation dose values of 2, 4, 6, 8, and 10 Gy by a ^60^Co unit. Their findings revealed that primary GBM-CD133 tumor initiating cells increased protein levels of phosphorylated STAT3 as well as showed high tumorigenic and radiochemoresistant properties. Moreover, they stated that treatment of GBM-CD133 cells with 100 µM resveratrol increased radiosensitivity and induced apoptosis by suppressing STAT3 signaling. Resveratrol also facilitated the differentiation of GBM-CD133^+^ into GBM-CD133^−^ and prevented the stemness gene signatures of GBM-CD133^+^. In addition, the xenotransplant experiments showed that the use of resveratrol can significantly improve the survival rate and synergistically increase the radiosensitivity of GBM-tumor initiating cells exposed to radiation [70]. It is notable that one of the main contributors to radioresistance are cancer stem cells (CSCs). These cells are also responsible for cancer progression and recurrence of gliomas after conventional treatment modalities [71–75]. It has also been reported that radioresistance is resulted from brain tumor-derived CD133^+^ cells that possess CSC capabilities [76–79].

The role of gap junction intercellular communication (GJIC) in the modification of growth and cell death has been proven. The changes in GJIC (including loss of homologous and/or heterologous) happens during the promotion/progression stages of carcinogenic process [80]; as the majority of cancerous cells lack GJIC [81]. Furthermore, additional epigenetic or genetic modifications, which stably inhibit GJIC, can lead to grow the cell without inhibition; hence, it becomes genomically unstable and obtains phenotypes needed for invasion and metastasis. In a study by Leone e al. [82], the regulation of cell cycle progression induced by resveratrol and association of this regulation with gap junction expression in human glioma U87 cells were investigated. They also evaluated the ability of this polyphenol to enhance radiosensitivity of these cancerous cells. In that study, the cells were treated with various dose values of resveratrol (0, 20, 40, 80, 160, 320, and 640 µM). To assess the combined therapeutic efficacy, the cells were treated with 20 mM resveratrol for 1 day and were then irradiated with 5 Gy X-rays. Their results showed that resveratrol significantly increased the fraction of cancerous cells in S phase of cell cycle in a dose-dependent manner (starting from 20 µM). Also, treatment with resveratrol resulted in a significantly higher fraction of cancerous cells in S phase compared to untreated cells (control group), with a concomitant significant decrease in the fraction of cancerous cells in G1 phase. These findings revealed a time-dependent manner and percentage of cancerous cells both in the S and G1 phase was comparable to that of control cells after ‏2-day resveratrol treatment. Additionally, 24–48 h after the X-ray treatment of cancerous cells, a significant increase of cancerous cells in the G2 phase compared to non-irradiated cells was observed, with a concomitant significant reduction of cancerous cells in G1 phase. The results of combined treatment (resveratrol + X-rays) demonstrated a significantly increase of cancerous cells in the S phase after irradiation, with a concomitant significant decrease in G1 phase cells (in a time-dependent manner). Compared to resveratrol or X-rays alone, the combined treatment showed a significant increase of S phase cells (after 28 and 30 h), with a concomitant decrease of G1 phase cells. Two days after irradiation, there was a significant increase in the fraction of G2 phase cells for combined treatment in comparison to single treatment. In conclusion, they stated that resveratrol can induce a delay in cell cycle progression and it is also capable of enhancement of GJIC, both alone and in combination with X-rays [82].

Hypoxia is known as a common characteristic of all solid malignancies [83–86] and it adjusts wide aspects of tumor biology, consisting of cellular proliferation, angiogenesis, invasion and metastasis [87, 88]. It has been shown that hypoxia is strongly related with poor prognosis [89–91] and it is considered as a leading cause of therapy resistance [92, 93]. Hypoxia-inducible factors (HIFs), including two α and β subunits, are mediators of hypoxia and responsible for monitoring cellular responses to oxygen levels [83, 94]. Among HIFs, HIF-1α and HIF-2α have critical roles in solid malignancies [83]; as these two factors adjust apoptosis, inhibit cellular differentiation, and activate DNA repairing enzymes, support formation of blood vessels, all of which are associated to treatment resistance [95]. Hypoxic tumor cells are also radioresistant [96] and these cells represent a two- to three-fold increase in radioresistance [97]. Hypoxia is a common characteristic in gliomas and decreases the sensitivity of cancerous cells to radiotherapy [98]; hence, inhibition of HIFs pathway may reduce radioresistance of glioma cells [99]. In this regard, it has been reported that resveratrol can inhibit HIF-1α expression in the hypoxia condition. Khoei et al. [100] investigated effect of resveratrol on radiosensitivity of iododeoxyuridine (IUdR), a halogenated pyrimidines analogue which is one of the most effective non-hypoxic radiosensitizers, in U87MG glioblastoma cell line. The cells were treated with 20 µM resveratrol and/or 1 µM IUdR, and were then exposed to 2 Gy radiation dose (by ^60^Co unit). Their findings revealed that resveratrol significantly decreased colony number and increased the DNA damages of GBM cells treated with IUdR in combination with radiotherapy. In conclusion, they stated that use of resveratrol (as HIF-1α inhibitor) in combination with IUdR (as radiosensitizer) can enhance the radiosensitization of U87MG glioblastoma cells [100].

In addition to the above-mentioned mechanisms, resveratrol can induce senescence and autophagy as well as attenuate the stemness of CSCs, leading to radiosensitization of cancer cells [101, 102]. Wang et al. [101] evaluated the therapeutic efficacy of resveratrol in combination with radiotherapy against radioresistant SU-2 glioma stem cells in both in vitro and invivo models. In that study, the cells and mice were treated with resveratrol in dose values of 75 µmol/L and 150 mg/kg, respectively and were then exposed to 0–6 Gy radiation dose values generated from a 6 MV X-ray linear accelerator. They represented that resveratrol has the ability to significantly increase the radiosensitivity of cancerous cells in both in vitro and nude mouse models which it can be attributed to its synergistic anti-cancer effects, including prevention of self-renewal and stemness, increase of apoptosis, induction of autophagy, and inhibition of DNA repair [101]. Self-renewal is a key property of stem cells and this ability of CSCs is necessary for tumorigenesis and tumor development [103]. CSCs also present stemness potential and this shows that proliferative cancer cells are continuously renewed by asymmetric division of CSCs [104].

### Therapeutic potentials of resveratrol in glioblastoma chemotherapy

A range from common chemotherapeutic agents are used for GBM treatment, such as temozolomide, doxorubicin, paclitaxel, etc. Some studies showed that resveratrol, as a chemosensitizer agent, can enhance the therapeutic efficacy of chemotherapeutic drugs through several mechanisms, which are discussed the below.

Oxidative stress conditions occur following the chemotherapy drug administration. The generated reactive oxygen species (ROS) can induce DNA damage either directly or indirectly, resulting in cancerous cell death [105]. It has been reported that resveratrol can increase ROS level in cancerous cells [106–108]. For instance, the interaction of resveratrol with the mitochondria of cancerous cells can induce an imbalance in cellular anti-oxidant activities, thereby a remarkable increase in the levels of both intracellular ROS and lipid peroxides [107]. Furthermore, resveratrol can inhibit oxidation–reduction (redox) system in cancerous cells [107]. It is notable that redox systems normally prevent cell oxidative damage; nevertheless, the cellular redox mechanisms in brain tumors are highly impaired which lead to the stimulation of survival cell pathways, thereby facilitating tumor growth and resistance [105]. Moreover, combined therapy using the resveratrol and chemotherapy agents showed synergistic effects against the cancerous cells in terms of ROS levels and redox activity [107, 108]. Öztürk et al. [108] investigated therapeutic efficacies of resveratrol (50 µM), paclitaxel (50 µM), and resveratrol plus paclitaxel on DBTRG glioblastoma cells. Their results showed that mitochondrial ROS levels significantly increased in these cancerous cells following treatment with paclitaxel and resveratrol. The mitochondrial ROS level of cells also increased further following combined treatment of paclitaxel plus resveratrol (synergistic effect). They also stated that synergic interactions of resveratrol on paclitaxel-induced oxidative stress can stimulate activation of the TRPM2 channel in the glioblastoma cells; as these changes contribute to the cancerous cell death by increment of the influx of Ca^2+^ into the cell through the channel [108].

Evasion of apoptosis is one of the features of most malignant cells, because defects in regulators of this physiological process invariably accompany tumorigenesis and maintain malignant progression [109]. Some chemotherapeutic agents induce apoptosis in cancerous cells [110]. It has also been reported that the resveratrol is able to induce apoptosis in different cancerous cells, such as glioma [111], prostate [112], breast [113], head and neck [114], ovarian [115] cancer cells. The apoptotic activity of resveratrol is related to induce ROS production, caspases activation, mitochondrial membrane permeability, p53 and BAX activation, etc. [106, 108, 116–118]. Furthermore, some studies have reported that the use of resveratrol can enhance chemotherapy-induced apoptosis in glioblastoma cell lines. The mechanisms would be either increased expression levels of apoptotic factors such as p53, BAX and caspase 3 or decreased expression levels of anti-apoptotic factors such as NF-κB and BCL-2 [119–122]. Li et al. reported that temozolomide-induced apoptosis in glioblastoma-initiating cells is enhanced by resveratrol through DNA double-stranded breaks (DSBs)/pATM/pATR/p53 pathway activation [119]. Huang et al. [120] stated that T98G glioblastoma cells receiving combination treatment of resveratrol and temozolomide had an increased apoptotic morphology, such as nuclear and cytoplasmic condensation and chromatin aggregation. Their other findings showed a significant increment in cleavage of caspase-3 and reduction in intracellular level and nuclear translocation of NF-κB in the cancerous cells treated with resveratrol and temozolomide than those treated with temozolomide alone [120]. Yang et al. reported that resveratrol could sensitize temozolomide-induced glioma cell apoptosis by suppressing Wnt signaling pathway activation and downregulating *O*-6-methylguanine-DNA methyltransferase expression [121].

Many studies have reported that resveratrol can regulate cell cycle progression and cell proliferation in different cancers, such as pancreatic cancer [123], breast cancer [124], melanoma [125], lung cancer [126], glioblastoma [59], and so on. Resveratrol exerts its tumor-suppressive effect through inhibition of NF-κB, mitogen-activated protein kinases (MAPKs), cyclooxygenases, metabolism of prostaglandins and also induction of apoptotic factors [12, 127, 128]. This chemosensitizer agent can also enhance AMPK expression (as a stimulator of p53) and prevent Akt expression (as a cancer proliferation gene) [63]. Inhibition of mTOR pathway may suppress Akt pathway [129]. Moreover, resveratrol may stop cell cycle progression through preventing DNA replication [130, 131]. It has also been reported that resveratrol via stimulation of SIRT1 increases the regulation of cyclin D1 which leads to inhibition of cancerous cell proliferation [132]. Furthermore, it has been reported that resveratrol can enhance cancer cell suppression induced by chemotherapy agents. Yuan et al. [133] stated combined treatment of resveratrol and temozolomide against U251 glioma cells significantly results in G2/M cell cycle arrest. They also reported that the chemotherapy drug in combination with resveratrol considerably increased ROS production which activated AMPK. Then, activated AMPK prevented mTOR signaling and downregulated BCL-2. Moreover, results of in vivo (an orthotopic xenograft model of glioblastoma) showed that combination treatment significantly decreased the volume tumor. Finally, they stated that resveratrol can enhance temozolomide-mediated anti-tumoral effects in glioblastoma through ROS-dependent AMPK–TSC–mTOR signaling pathway [133].

**Fig. 2:**
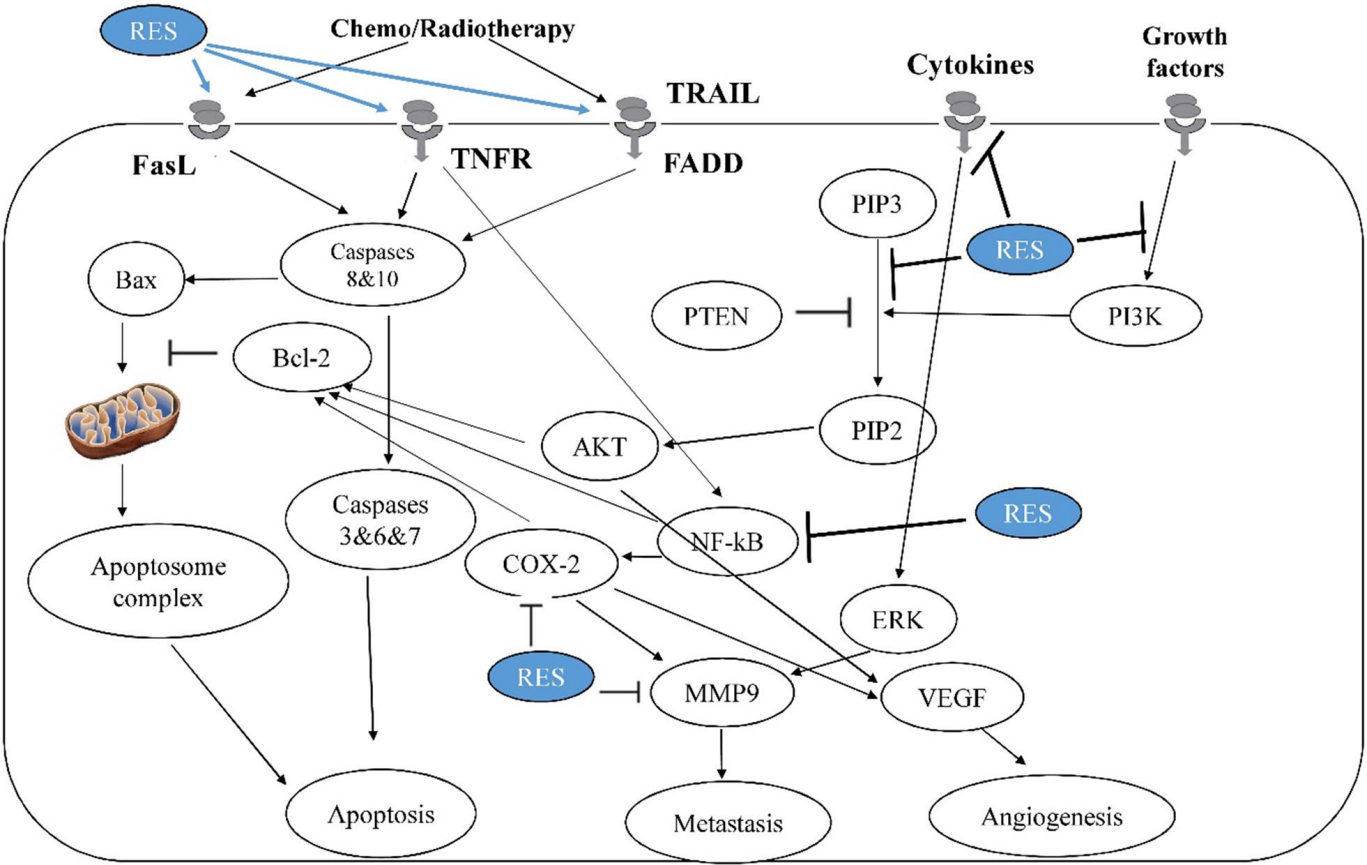
Resveratrol as an enhancer of radio/chemosensitizer. Resveratrol is able to induce or inhibit various pathways related to apoptosis, angiogenesis and metastasis. This figure shows some signaling pathways in cancer (stem) cells that regulate apoptosis, metastasis or angiogenesis following radiotherapy or chemotherapy. Resveratrol can potentiate apoptosis signaling pathways, while it suppresses angiogenesis and metastasis

CSCs, a small population of cancerous cells, are tumor-initiating cells and include a group of quiescent self-renewing cell types which pre‐exist in primary malignant tumors and localized within the tumor niches bearing enriched functional potential to drive tumor growth, to reconstruct their heterogeneity and to make changes in tumor regenerative capacity [134, 135]. The different cell surface markers are often applied to identify and enrich CSCs, including CD44, CD24, and CD133 [136]. Glioblastoma stem cells play main roles in the glioblastoma development and therapeutic resistance [76, 137–139]. It has also been reported that there is an association between tumor formation by the glioblastoma stem cells and their peculiar resistance to chemotherapy treatment in comparison with other cell populations of the tumor [140]. The first accepted surface marker for glioblastoma stem cells was CD133 [141] that it allows the subdivision of stem cells into two groups of CD133^+^ or cancer stem cells and CD133^−^ or non-cancer stem cells [142]. Other markers expressed in glioblastoma stem cells are CD15, CD184, CD44, A2B5, CD90, SOX2, OCT-4, SALL4, NANOG, ALDH1, L1CAM, KLF4, and so on [76, 143–153]. Some studies have reported that resveratrol is able to target glioma glioblastoma stem cells through various molecular pathways involved in self-renewal and several stem cell markers. Song et al. showed that resveratrol could inhibit self-renewal ability of glioma stem cells and cancer stem cell markers (such as Bmi1 and Sox2) induced by epithelial mesenchymal transition [154]. Sayd et al. reported that increased expression of SIRT2 induced by resveratrol inhibits glioma stem cell proliferation [155]. Clark et al. [59] mentioned that resveratrol significantly inhibited proliferation and invasion of glioblastoma stem cells. This chemosensitizer agent also inhibited the sphere-forming ability. They also reported that resveratrol decreases AKT phosphorylation and induces p53 expression and activation. It is noteworthy that AKT and p53 mechanisms involve in growth, survival, and invasion of glioblastoma [59]. Cilibras et al. evaluated effect of resveratrol on glioma stem cells and showed that it is able to inhibit cell proliferation, increase cell mortality, and decrease cell motility. They also reported that resveratrol can modulate Wnt signaling pathway and epithelial mesenchymal transition activators [156]. Li et al. [119] demonstrated the use of resveratrol sensitizes temozolomide-induced apoptosis of glioblastoma-initiating cells through activation of the DSBs/pATM/pATR/p53 pathway. They also mentioned that combined treatment of temozolomide and resveratrol could induce cell differentiation and inhabit self-renewal capacity of glioblastoma-initiating cells via STAT3 inactivation [119]. Figure 2 represents some signaling pathways in cancer (stem) cells that regulate apoptosis, metastasis or angiogenesis following radiotherapy or chemotherapy.

**Fig. 3:**
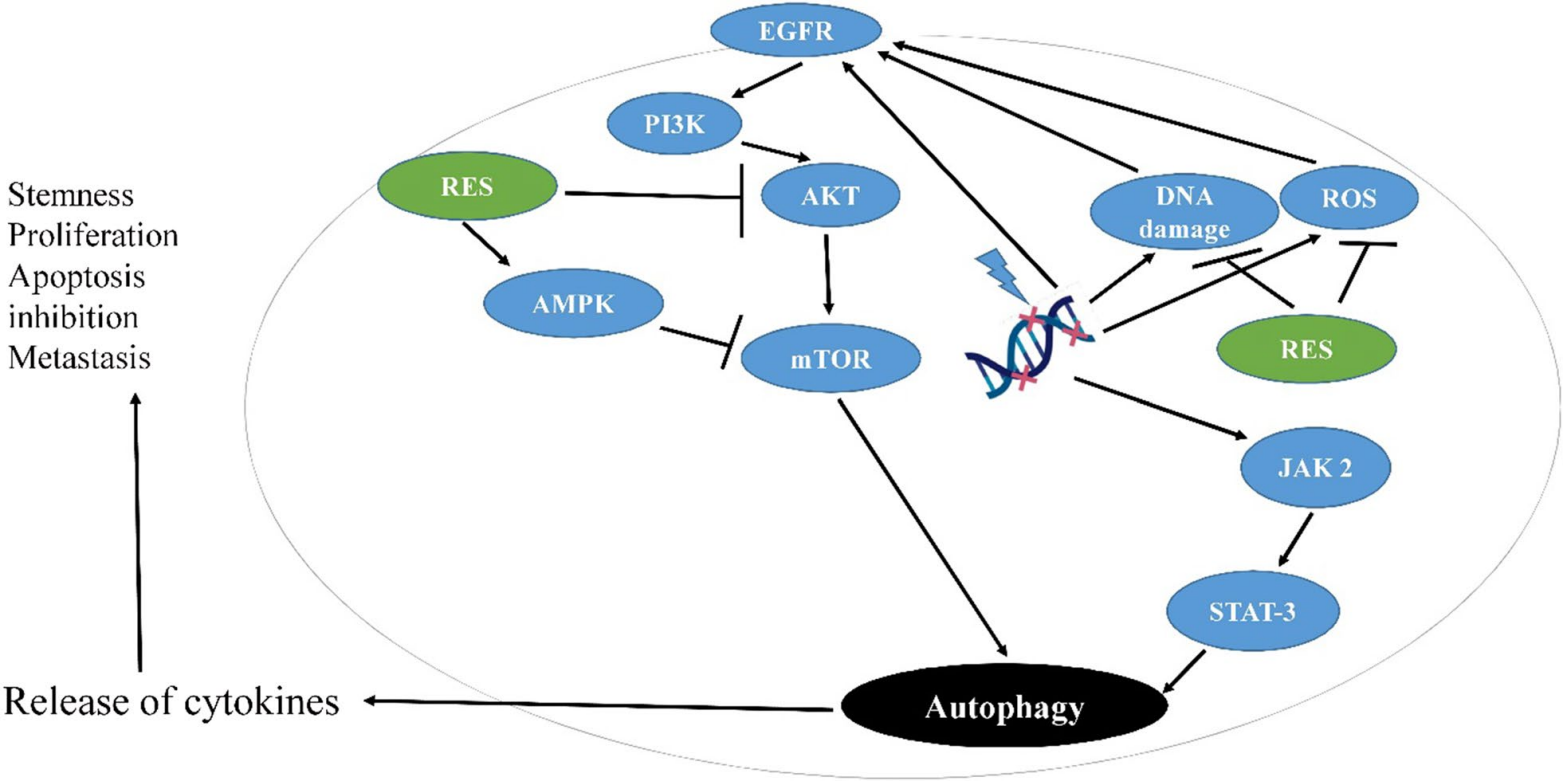
Schematic mechanisms for anti-tumor effect of resveratrol in glioblastoma through inhibition of autophagy pathways

Autophagy, as a physiological cellular process, participates in cell death under different conditions [157, 158]. The autophagic process is also associated with various diseases such as cancer [159]. It can decrease cell instability and damage to prevent tumorigenesis; hence, the regulation of autophagic process is of great significance in cancer treatment [160, 161]. Aberrant regulation of autophagy has been reported in different diseases and is common in tumors [159, 160]. For instance, autophagy deficiency can result in aberrant accumulation of p62 (an autophagy adaptor protein and preferred target for autophagy) and activate p62-regulated pathways, such as activation of mTOR and Keap1–Nrf2 pathways which are associated with tumor development [160]. Moreover, some cancer types such as glioblastoma are intrinsically resistant to apoptotic cell death and may be more sensitive autophagy [162]. Autophagy has a dual role in cancers and may result in sensitization or resistance of cancerous cells. This dual role induced by autophagy is remarkably dependent on genetic changes in cancerous cells [12]. The chemotherapeutic agents can induce autophagy in cancerous cells through different mechanisms; however, alteration in autophagic response could be considered as a key mechanism in drug resistance [163–166]. Resveratrol regulates the autophagic process and may affect the response of cancerous cells to therapy [12]. It has also been reported that resveratrol inhibits glioblastoma cell growth and causes the cell death through mechanisms involved in autophagy [167, 168]. Induction of autophagy by resveratrol can happen by several mechanisms, including the acceleration of p62 degradation, suppression of mTOR and Nrf2 activations, induction of apoptosis, activation of p38-MAPK pathway, inhibition of STAT3 activation, and so on [160, 169–171]. Furthermore, the effect of resveratrol on chemotherapy-induced autophagy in glioblastoma cells has been investigated (Fig. 3). Lin et al. [172] reported that temozolomide induced both apoptosis and cytoprotective autophagy in glioma cells via a ROS burst and extracellular signal-regulated kinase (ERK) activation; however, resveratrol suppressed them and resulted in a reduction in autophagy and an increment in apoptosis. Therefore, they suggested that the ROS/ERK pathway can play a critical role in the fate of cancerous cells after temozolomide treatment. Furthermore, an in vivo mouse xenograft study revealed that the combined treatment of temozolomide and resveratrol can decrease ERK activity and LC3-II protein level as well as increase the cleavage of PARP; as these findings represented that resveratrol enhances tumor apoptosis through suppressing the autophagic pathway. In conclusion, they reported that resveratrol sensitizes glioma cells to temozolomide-mediated apoptosis in a synergistic manner via down-regulation of protective autophagy [172]. Zanotto-Filho et al. [164] showed that temozolomide efficacy in glioblastoma treatment improved by inhibition of ERK1/2-dependent autophagy induced by resveratrol. In another study by Filippi-Chiela et al. [163], it was shown that resveratrol increases autophagy induced by temozolomide in glioblastoma cells, but autophagy did not affect acute cell death.

## Resveratrol derivatives/analogues in glioblastoma treatment

In the current study, the anti-tumoral, radiosensitizer, and chemosensitizer properties of resveratrol were reviewed and it was found that resveratrol, in addition to having anti-tumoral properties alone, can have synergistic effects in combination with radiotherapy and chemotherapy. Despite its remarkable anti-cancer beneficial effects, unfavorable pharmacokinetics/pharmacodynamics profile of resveratrol such as poor bioavailability has restricted its applications. Hence, researchers have synthesized novel derivatives and analogues for this anti-tumoral agent using various modification strategies to overcome these restrictions and improve anti-tumoral efficacy. The anti-tumoral properties of resveratrol derivatives/analogues have been evaluated in different cancers such as breast cancer [173], ovarian cancer [174], gastric cancer [175], renal carcinoma, lung cancer, colon cancer, prostate cancer [176], glioblastoma [177], etc. The design, synthesis, and anti-tumoral properties of resveratrol-based compounds have recently reviewed by Ahmadi and Ebrahimzadeh [178].

In a study by Chelsky et al. [177], anti-tumoral effect of the resveratrol derivative (*E*)-4-(3,5-dimethoxystyryl)phenyl acetate against U251MG glioma cells was investigated. Their findings showed that the use of this resveratrol analog resulted in reduction of colony formation, induction of cell cycle arrest in the G2/M phase, suppression of survivin, Bcl-xL, cyclin D1 and cyclin B1 expression, and induction of cleavage of caspases 3, 8, and 9 and poly(ADP ribose) polymerase. Mechanistically, it was found that treatment of U251MG cells with this resveratrol analog resulted in suppression of STAT3 tyrosine705 phosphorylation and induction of STAT3 serine727 phosphorylation [177]. Zielińska-Przyjemska et al. [179] assessed effects of resveratrol and its analogs (3,5,4′-Trimethoxystilbene (TMS) and pterostilbene) on apoptosis and cell cycle in rat C6 and human T98G glioma cells. Their results showed that resveratrol and pterostilbene administrations increased percentage of the cancerous cells in S phase, while TMS led to a massive accumulation of cancerous cells at the G2/M phase of the cell cycle. Furthermore, the apoptosis rate in the cancerous cells was most significantly increased by TMS through p53 induction [179]. Majchrzak-Celinska et al. [180] investigated the anti-tumoral effects of resveratrol and its five analogs on T98G glioblastoma cells. They reported that these agents downregulated the expression of genes involved in Wnt/β-catenin pathway. Moreover, it was observed that the 4′-methoxy substituted derivatives had higher activity, whereas 3,4,4′-Tri-methoxy-trans-stilbene was the most potent Wnt/β-catenin pathway inhibitor. Furthermore, administration of the compounds did not affect DNA methylation level of MGMT, SFRP1, or RUNX3T, despite moderate alterations in expression levels of epigenetic modifiers DNMT3B and TET1-3 were observed. Importantly, it was found that treatment with 3,4,4′-Trimethoxy-trans-stilbene and 3,4,2′,4′-tetra-methoxy-trans-stilbene resulted to cycle arrest in the S phase and induced apoptosis [180].

To the best of our knowledge, no research has been conducted on the radio/chemosensitizer properties of resveratrol derivatives/analogues in glioblastoma treatment. Therefore, it is proposed studies geared towards this direction.

## Delivery systems of resveratrol in glioblastoma treatment

Besides the beneficial effects of resveratrol, several drawbacks such as poor solubility in water, high photosensitivity, and low oxidative stability have limited its application. It has been reported that nanostrategies for delivery of resveratrol can overcome to these limitations. For instance, improved toxicity against cancerous cells was obtained by polymeric and lipid-based nanocarriers. Or, silica nanoparticles significantly improved the biological activity and loading capacity of resveratrol and gold and silver nanoparticles promoted anti-bacterial and anti-tumoral activities of resveratrol [181].

There are some studies which have evaluated the efficacy of delivery systems of resveratrol in glioblastoma treatment. Shao et al. [182] synthesized resveratrol-loaded methoxy poly(ethylene glycol)-poly(caprolactone) (mPEG-PCL) nanoparticles with high encapsulation efficiency, because of its lipophilicity. They also stated that the resveratrol-loaded nanoparticles at lower concentration could cause significantly higher glioma cell death in comparison with equivalent dose of free resveratrol. Moreover, ROS determination showed the significantly lower intracellular ROS levels in resveratrol-treated cancerous cells compared to nanoparticle-treated cancerous cells. Hence, they reported that the differential cytotoxicity between free resveratrol and resveratrol-loaded nanoparticles may be mediated by the difference of intracellular ROS levels [182]. Figueiró et al. [183] investigated the anti-tumoral effect of resveratrol-loaded lipid-core nanocapsules against C6 glioma cells in both in vitro and in vivo models. In vitro, the resveratrol-loaded nanoparticles reduced the viability of cancerous cells to a higher extent than free resveratrol through induction of apoptotic cell death. In vivo, treatment with the nano-complex promoted a remarkable reduction in tumor size and also decreased the incidence of some cancer-associated characteristics (such as intratumoral edema and hemorrhaging) compared to free resveratrol [183]. Guo et al. [184] assessed therapeutic efficacy of transferrin-modified PEG-poly lactic acid (PLA) nanoparticles conjugated with resveratrol against C6 and U87 glioma cells in both in vitro and in vivo models. Their findings showed that in vitro cytotoxicity of nano-complex against the cancerous cells was higher than that of free resveratrol. In comparison with free resveratrol, the nano-complex could significantly reduce tumor volume and accumulate in brain tumor, thereby prolonging the survival of tumor-bearing rats [184]. Jhaveri et al. [185] synthesized resveratrol-loaded PEGylated liposomes and modified the liposome surface with transferrin moieties to make them tumor cell-specific. They reported that the transferrin-modified PEG-PLA nanoparticles conjugated to resveratrol had significantly more cytotoxic and increased apoptosis accompanied by activation of caspases 3/7 in U-87 MG glioblastoma cells compared to free resveratrol or resveratrol-loaded PEGylated liposomes. In addition, their results demonstrated that transferrin-modified PEG-PLA nanoparticles conjugated to resveratrol were more effective than other treatments in inhibition of tumor growth and improvement of survival in tumor-bearing mice [185]. Xu et al. [186] constructed, characterized and tested mPEG-PCL nanoparticles coloaded with temozolomide and resveratrol for anti-tumoral effect against U87 glioma cells in both in vitro and in vivo models. The temozolomide/resveratrol-coloaded nanoparticles induced higher apoptosis in the cancerous cells compared to those treated by the combination of free temozolomide and resveratrol. Moreover, the temozolomide/resveratrol-coloaded nanoparticles led to more effective inhibition of phosphor-Akt, resulting to upregulation of the downstream apoptotic proteins. Furthermore, the in vivo findings demonstrated the superior tumor delaying effect of the temozolomide/resveratrol-coloaded nanoparticles compared to that of free temozolomide and resveratrol combination [186].

However, more studies are required to approve the effectiveness of nano-based delivery systems of resveratrol in glioblastoma treatment. In addition, it is proposed to evaluate the delivery systems of resveratrol in combination with radiotherapy and chemotherapy.

## Conclusions

The standard treatment modality for GBM include surgery, radiation therapy and/or chemotherapy. Nevertheless, these cancerous cells are resistant to radiation therapy and chemotherapy; hence, efficient therapeutic modalities for GBM treatment are still required. Resveratrol, as an anti-tumoral agent, has shown to be effective in GBM treatment and it exerts this property through various mechanisms such as regulation of cell cycle progression and cell proliferation, autophagy, oxidant system, apoptosis pathways, etc. The synergistic effects of resveratrol in combination with radiation therapy (radiosensitizer) and chemotherapy (chemosensitizer) have also been confirmed. Furthermore, the use of derivatives/analogues and delivery systems of resveratrol could improve anti-tumoral efficacy of resveratrol.

## Data Availability

The datasets used and/or analyzed during the current study are available from the corresponding author on reasonable request.
